# The Effect of Recombinant Human TSH on Sclerostin and Other Selected Bone Markers in Patients after Total Thyroidectomy for Differentiated Thyroid Cancer

**DOI:** 10.3390/jcm10214905

**Published:** 2021-10-24

**Authors:** Arkadiusz Zygmunt, Kinga Krawczyk-Rusiecka, Elżbieta Skowrońska-Jóźwiak, Katarzyna Wojciechowska-Durczyńska, Ewa Głowacka, Zbigniew Adamczewski, Andrzej Lewiński

**Affiliations:** 1 Department of Endocrinology and Metabolic Diseases, Medical University of Lodz, 93-338 Lodz, Poland; arkadiusz.zygmunt@umed.lodz.pl (A.Z.); kinga.krawczyk-rusiecka@umed.lodz.pl (K.K.-R.); elzbieta.skowronska-jozwiak@umed.lodz.pl (E.S.-J.); katarzyna.wojciechowska-durczynska@umed.lodz.pl (K.W.-D.); 2 Department of Laboratory Diagnostics, Research Institute, Polish Mother’s Memorial Hospital, 93-338 Lodz, Poland; ewa.biol@interia.pl; 3 Department of Endocrinology and Metabolic Diseases, Research Institute, Polish Mother’s Memorial Hospital, 93-338 Lodz, Poland; zbigniew.adamczewski@umed.lodz.pl

**Keywords:** thyrotropin, sclerostin, parathormon, bone turnover markers

## Abstract

The direct effect of TSH on bone metabolism in vivo is difficult to capture as the changes of its concentrations are followed by respective alterations of thyroid hormone levels. We evaluated the effect of recombinant human TSH (rhTSH) on sclerostin and other bone markers in 29 patients after total thyroidectomy for differentiated thyroid cancer (DTC), without any signs of disease recurrence, who received L-thyroxine, most at non-suppressive doses. For two consecutive days, the patients were administered a standard dose of 0.9 mg rhTSH, i.m. Concentrations of sclerostin, osteocalcin, β-CrossLaps, PTH, and some other parameters, were measured before and five days after the first rhTSH administration. The greater the increase in TSH concentration (∆TSH), the greater the decrease in: ∆sclerostin (r = −0.672; *p* < 0.001), ∆β-CrossLaps (r = −0.580; *p* < 0.001) and ∆osteocalcin (r = −0.405; *p* = 0.029) levels, were recorded. The degree of TSH increase depended on the baseline PTH (r = 0.651; *p* < 0.001), age, and creatinine concentrations. rhTSH strongly inhibited bone turnover, thus, TSH—independently of thyroid hormones—exerted a direct protective effect on bone metabolism. Baseline PTH affected the magnitude of TSH increase and the degree of lowering in sclerostin and β-CrossLaps that suggest factors affecting PTH may play a role in the effect of TSH on the bone.

## 1. Introduction

Since Inoueet et al. [1] and Abe et al. [2] have demonstrated the presence of TSH receptors in osteoblasts and osteoclasts, the potential role of TSH in bone remodeling has been considered. TSH receptors are also present in chondrocytes [3], but they have not been found on osteocytes, so far. Most studies indicate that TSH inhibits osteoclast differentiation and function. Moreover, it is postulated that TSH has an inhibitory effect on chondrocytes and osteoblasts, as well [4]. Therefore, TSH inhibits bone metabolism and thus plays a protective role for the bone [1,5].

Sclerostin is a 22-kDa protein that is a product of the SOST-gene, which is a well-known negative regulator of bone formation [6,7,8]. It has been shown that sclerostin: (1) inhibits proliferation and differentiation of pre-osteoblastic cells, as well as decreases activation of mature osteoblast; (2) stimulates bone resorption and decreases mineralization; (3) increases apoptosis of the osteogenic cells; (4) maintains bone lining cells in their quiescent state; and (5) regulates osteocyte maturation and osteocytic osteolysis [9].

Factors that lead to an increase in sclerostin secretion include oestrogen deficiency [10] and mechanical unloading [11]. It was also noticed that in conditions of thyroid hormones excess, the levels of sclerostin are higher than after the treatment of hyperthyroidism [12]. On the other hand, the excess of PTH resulted in the reduction of sclerostin [13]. Thus, a reduction in sclerostin concentration contributes to an increase in bone mineral density (BMD) [14], and anti-sclerostin monoclonal antibodies are currently used in the treatment of osteoporosis [15].

Alterations in TSH concentrations can be secondary or primary. The most common are secondary changes which reflect the adequate response of the healthy pituitary gland to too high or too low levels of thyroid hormones in the body. Excess of thyroid hormones (resulting from hyperthyroidism or thyrotoxicosis caused by destructive thyroiditis or therapy with supraphysiological doses of L-T4) is accompanied by TSH suppression, while thyroid hormone deficiency (in the course of hypothyroidism) is accompanied by an increase in TSH concentration.

Disorders in the course of which TSH changes occur primarily are relatively rare and are caused by a disease of the pituitary gland. Since TSH is a physiological stimulator of the thyroid growth and activity, its excess (in e.g., TSH-secreting pituitary tumors), as well as its deficiency (hypopituitarism) lead to an increase (secondary hyperthyroidism) or a deficit (secondary hypothyroidism) of thyroid hormones, respectively, which again affects bone metabolism [16].

Typical of these situations, regardless of the pathogenesis and frequency, is the coexistence of both TSH and thyroid hormones changed levels, which makes it very difficult to distinguish between the effect of thyroid hormones and the influence of TSH on bone metabolism.

To avoid such ambiguities, one should study such a clinical situation in which the concentrations of thyroid hormones are relatively constant and predictable. Such conditions were ensured in our study. There was virtually an absence of the thyroid gland in our studied group [patients after thyroidectomy for differentiated thyroid carcinoma (DTC), very low thyroglobulin (Tg) concentration, indicating either no or only a small amount of thyroid tissue] and L-T4 was administrated in constant doses dependent on the risk of DTC recurrence, mostly non-suppressive doses [17].

In addition, the increase in TSH was very significant (TSH increased to a concentration value corresponding to overt primary hypothyroidism) and acute (rhTSH was administered only for two days). The above conditions are the basis for investigating the real effect of rhTSH on bone metabolism in humans in vivo.

The aim of the study was to evaluate the effect of recombinant human TSH (rhTSH) on sclerostin, other selected bone markers, and parameters of calcium-phosphate homeostasis in vivo.

## 2. Materials and Methods

29 patients (26 women and 3 men) aged 52.4 ± 13.9 years, hospitalized at the Department of Endocrinology and Metabolic Diseases, Medical University of Lodz, were examined. The study was conducted according to the guidelines of the Declaration of Helsinki and approved by the Ethics Committee of the Polish Mother’s Memorial Hospital—Research Institute, Lodz, Poland (approval code no.: 58/2011, approval date: 14 December 2011).

Patients had a history of surgical treatment for papillary (*n* = 28) or follicular (*n* = 1) thyroid cancer and then, depending on the stage of the cancer, some of them (*n* = 23) received radioiodine (^131^I) ablation treatment.

All patients were taking L-T4, either in substitutive doses (*n* = 18) or in doses aimed at partial (incomplete) TSH suppression (*n* = 11; TSH target: 0.1–0.4 mIU/L).

No clinical or biochemical features of recurrence of disease were found in any of the subjects. The characteristics of the patients included in the study are presented in Table 1.

All patients had a normal parathyroid function. None of the subjects had been treated earlier for metabolic diseases (including osteoporosis). Nobody smoked. The exclusion criterion was treatment with drugs known to influence bone metabolism during the previous 24 months before the enrolment.

The administration of rhTSH was part of the routine diagnostics carried out in all patients, the aim of which was the evaluation of recurrence of disease without L-T4 therapy withdrawal.

Each patient received a standard dose of 0.9 mg rhTSH (Thyrogen, Sanofi-Genzyme), i.m. for two consecutive days according to the standard protocol.

Blood samples were drawn from each patient after overnight fasting between 08.00 and 09.00 a.m., before (point 0) and five days after the first rhTSH administration (point 5).

All samples were centrifuged and stored at −70 °C until determinations were performed. All measurements were performed in the same assay.

Sera were assayed for sclerostin, osteocalcin, crosslinked isomerized type I collagen fragments (β-CrossLaps), TSH, free thyroxine (FT4), free triiodothyronine (FT3), PTH, and 25-hydroxyvitamin D [25(OH)D] (only at point 0).

Sclerostin was measured by a quantitative two-site immunometric (sandwich) assay, using the enzyme-linked immunoassay (ELISA) method (Biomedica, Vienna, Austria).

Osteocalcin, β-CrossLaps, TSH, FT4, FT3, PTH, 25(OH)D and Tg were determined by commercially available two-site immunometric assay, using electrochemiluminescence detection (Cobas e 601 or Cobas e 411—Roche, Basel, Switzerland).

Serum calcium, phosphate, and creatinine and the excretion of calcium and phosphorus in the 24-h urine collection were determined as well, using a routine analytical method, before (point 0) and on the fifth day after the first dose of rhTSH administration (point 5). Glomerular filtration rate (GFR) was estimated by MDRD 4-Variable Equation [18].

### Statistical Analysis

Values are expressed as mean ± SD. The data were statistically analyzed, using a non-parametric test for dependent groups (Mann–Whitney Rank Sum test), Kruskal–Wallis One Way Analysis of Variance on Ranks, followed by Dunn’s test and Pearson Correlation. In all analyses, statistical significance has been considered achieved at a value of *p* < 0.05.

Data processing, statistical analyses, and figures were performed by using SigmaPlot 12.3 (Systat Software, Inc, San Jose, CA, USA).

## 3. Results

Table 2 shows the obtained test results.

Apart from a significant increase in TSH concentration after administration of rhTSH (28.62 ± 16.41 vs. 0.52 ± 1.31 mIU/L; *p* < 0.001), no other differences were observed between two time points (point 5 vs. point 0). Interestingly, we observed that the increase in TSH value in each patient [∆TSH = TSH (point 5) − TSH (point 0)] was dependent on age, which corresponded with the decrease of creatinine clearance (r = −0.444, *p* = 0.016) and PTH before rhTSH administration (r = 0.651, *p* < 0.001). The older the patient was, the greater the ∆TSH was (r = 0.701; *p* < 0.001).

**Figure 1:**
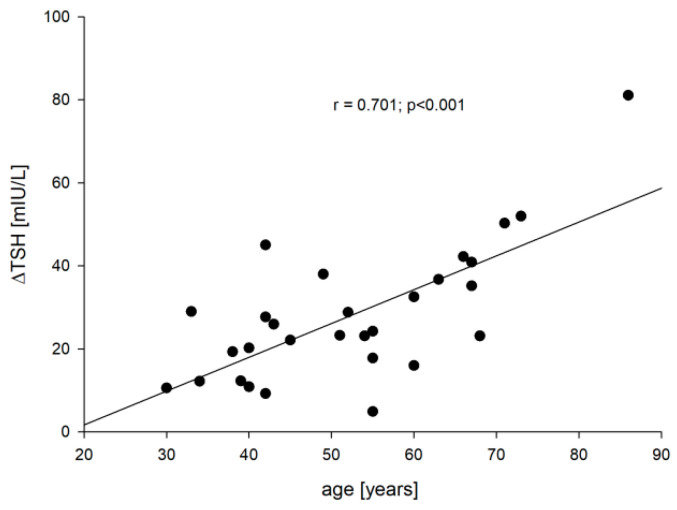
The correlation between the age vs. ∆TSH.

Figure 1 shows the correlation between the ∆TSH depending on age. Such a correlation was not observed depending on BMI and the baseline TSH concentration (point 0), as well as other markers of bone turnover apart from sclerostin. In this case, we found a correlation between age and sclerostin at point 0 (r = 0.448, *p* = 0.015) and between age and ∆sclerostin (r = −0.536, *p* = 0.003).

**Figure 2:**
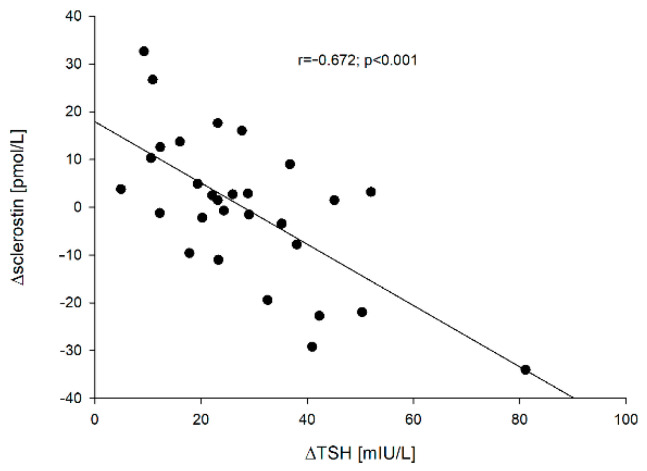
The correlation between the ∆TSH vs. ∆sclerostin.

**Figure 3:**
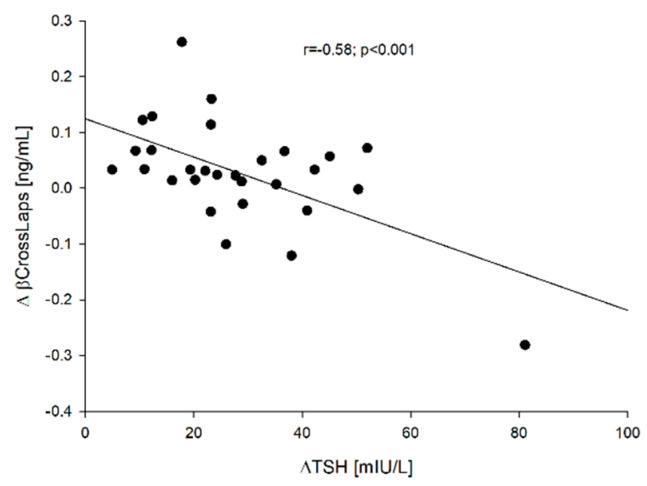
The correlation between the ∆TSH vs. ∆β-CrossLaps.

**Figure 4:**
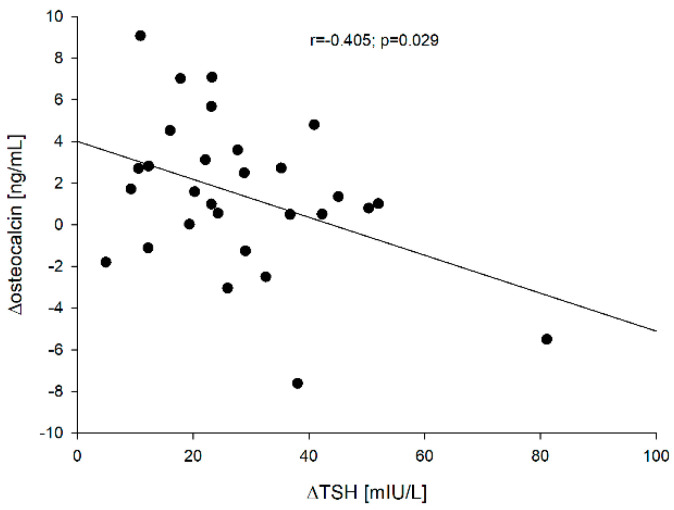
The correlation between the ∆TSH vs. ∆osteocalcin.

We captured negative correlations between ∆TSH vs. ∆sclerostin [=sclerostin (point 5) − sclerostin 0] (r = −0.672; *p* < 0.001), ∆TSH vs. ∆β-CrossLaps [=β-CrossLaps (point 5) − β-CrossLaps (point 0)] (r = −0.580; *p* < 0.001) and ∆TSH vs. ∆osteocalcin [=osteocalcin (point 5) − osteocalcin (point 0)] (r = −0.405; *p* = 0.029). These correlations are presented in Figure 2, Figure 3 and Figure 4, respectively.

**Figure 5:**
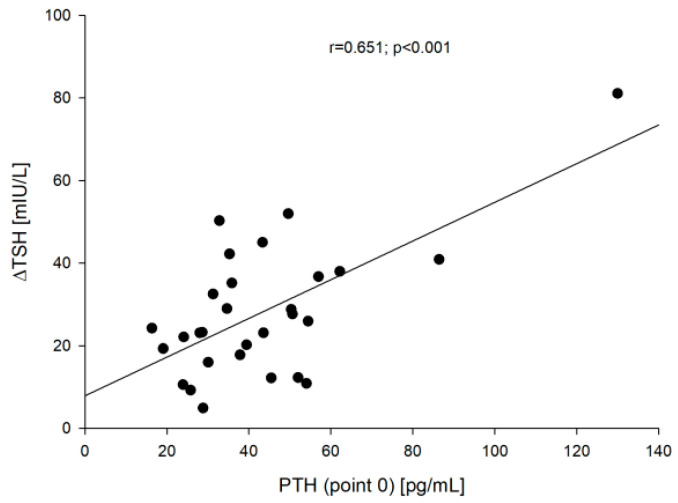
The correlation between the PTH (point 0) vs. ∆TSH.

**Figure 6:**
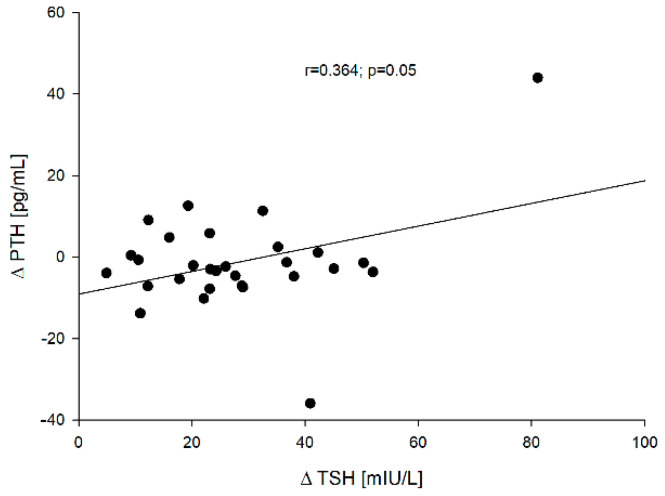
The correlation between the ∆TSH vs. ∆PTH.

On the other hand, a significant positive correlation was found between PTH vs. ∆TSH concentration before (point 0) (r = 0.651; *p* < 0.001) and after rhTSH administration (point 5) (r = 0.681; *p* < 0.001) and ∆TSH vs. ∆PTH [=PTH (point 5) − PTH (point 0)] (r = 0.364; *p* = 0.05). These correlations are presented in Figure 5 [PTH (point 0) vs. ∆TSH] and Figure 6 (∆TSH vs. ∆PTH).

**Figure 7:**
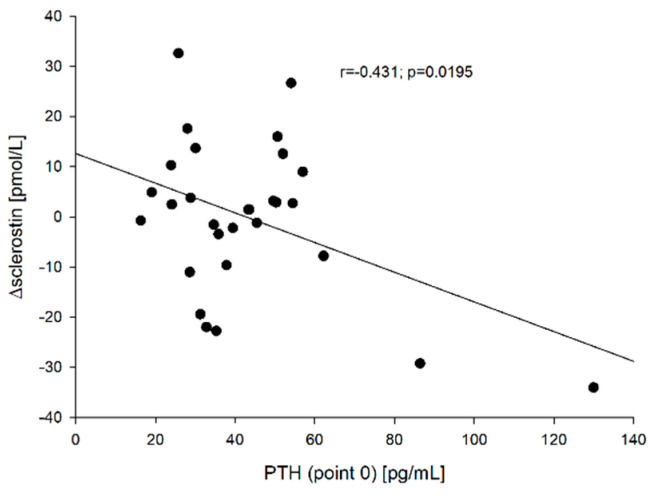
The correlation between the PTH (point 0) vs. ∆sclerostin.

**Figure 8:**
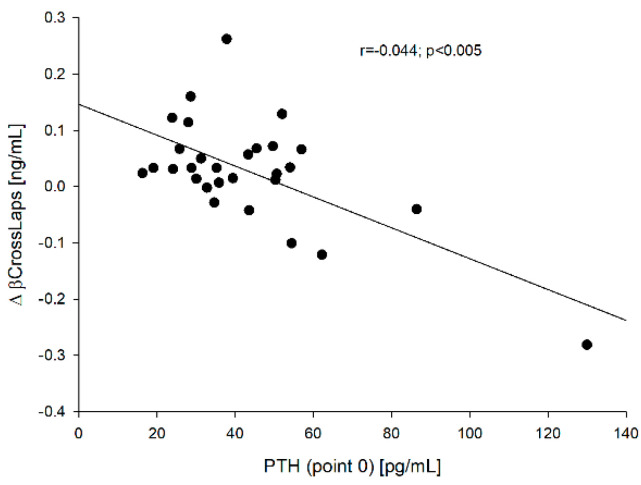
The correlation between the PTH (point 0) vs. ∆β-CrossLaps.

Moreover, the relationships between PTH concentration [both before (point 0)—Figure 7 and after rhTSH administration (point 5)] and ∆sclerostin (r = −0.431, *p* = 0.0195 and r = −0.405, *p* = 0.0293, respectively), and between PTH at point 0 and ∆β-CrossLaps (r = −0.644, *p* < 0.005)—Figure 8 were found.

## 4. Discussion

### 4.1. Effect of rhTSH on Sclerostin and Bone Turnover Markers

The very idea of examining the influence of rhTSH on bone metabolism in patients with postoperative hypothyroidism in the course of well-differentiated thyroid cancer is not new [19,20,21,22]. For the first time, however, we examined the influence of rhTSH on sclerostin and, apart from the classic analysis of results (comparison of values in particular groups), we also proposed an analysis of changes in markers in individual subjects.

In the analysis of two dependent groups (before and after drug administration), no significant changes were observed (apart from an understandable significant increase in TSH). In such an analysis, it is very difficult to show differences, because high inter-individual variability is a characteristic feature of bone turnover markers [23]. This is one of the reasons why, as well as due to low sensitivity and specificity, markers of bone turnover have never gained recognition in the diagnosis of osteoporosis [24] but have a recognized role in monitoring the treatment of osteoporosis (analysis of markers concentration during treatment versus baseline values) [25]. Therefore, the study analyzed how the concentrations of sclerostin (∆sclerostin) and other examined markers of bone turnover (∆β-CrossLaps, ∆osteocalcin and ∆PTH) change in individual subjects, taking into account the degree of increase in TSH (∆TSH).

Despite the relatively small (*n* = 29) and age-diversified (x ± SD: 52.4 ± 13.9; min-max: 30–86 years) group of patients, it has been proven that the increase in TSH significantly inhibits bone metabolism. The greater the increase in TSH is, the greater the changes in bone markers are (Figure 2, Figure 3 and Figure 4). The *p-value is small, while the correlation indices *point to a high correlation. The obtained results are consistent with the results of in vitro studies [26], but in humans, it varies. Martini et al. [19] who mainly studied the influence of rhTSH on the OPG (osteoprotegerin)/RANKL (receptor of nuclear factor-κB ligand)/RANK (receptor of nuclear factor-κB) system, found in postmenopausal women an increase in the serum concentration of N-terminal propeptide of type-I procollagen (PINP), marker of bone formation. Mazziotti et al. [22] noticed a decrease in CrossLaps concentration on day two after administration of rhTSH in postmenopausal women and an increase in bone alkaline phosphatase (BALP) concentration on days two and seven after administration of rhTSH compared to the baseline values in postmenopausal women. Therefore, both teams of researchers observed an increase in bone markers reflecting bone formation. They failed to capture the effect of rhTSH administration on OPG. Karga et al. [20] observed a significant reduction in the urinary excretion of N-terminal telopeptide of type I collagen (U.NTx) and urinary excretion of C-terminal telopeptide of type I collagen (U. CTx) on day 1 in relation to baseline values and when compared to those patients who did not receive rhTSH, which would be consistent with our observations, i.e., generalized inhibition of bone turnover. On the other hand, Iakovou et al. [21] found no statistical differences in the examined markers of bone turnover. It should be emphasized once again that all these researchers analyzed the parameters between particular groups, not individual patients, while the analysis was also carried out with the control group.

So far it is known that sclerostin is produced mainly in osteocytes, and there have been no TSH receptors found on them up to now. However, in our study, the greatest observed correlation (when analyzing the r value) was the negative correlation between TSH and sclerostin concentration. This would suggest a direct effect of TSH on osteocytes or a high efficiency of the indirect influence of TSH on sclerostin synthesis by acting on osteoblasts and osteoclasts.

### 4.2. Relationships between PTH and TSH and Sclerostin

The analysis of factors determining the increase in TSH (∆TSH) produced interesting results.

It could be assumed that (1) since each patient was administered the same dose of the drug (0.9 mg rhTSH i.m. for two consecutive days), the increase in TSH in subjects with lower body mass index (BMI) should be greater than in obese subjects (see BMI values of the study group—Table 1 and (2) if the patient had lower TSH at baseline, the increase will be greater.

In our observation, these assumptions were not confirmed either for BMI (r = 0.249; *p* = 0.192) or for baseline TSH (r = 0.169; *p* = 0.38). However, the increase in TSH strongly depended on age (r = 0.701; *p* < 0.001), on creatinine (r = 0.467; *p* = 0.011) and on the baseline PTH concentration, which corresponded to the captured correlation between PTH0 vs. creatinine (r = 0.485; *p* = 0.008) and age (r = 0.42; *p* = 0.023). On the other hand, the correlation found between ∆PTH and ∆TSH was much weaker and was characterized by a much higher *p* value than in the case of sclerostin or other investigated markers. As if baseline PTH determined the increase in TSH, but the effect of TSH changes was not as significant on PTH changes as it was for other molecules tested. This can be explained by the fact that the effect of TSH increase on PTH changes is indirect and is due either to changes in serum calcium or phosphorus levels. We did not observe such relationships (parameters were determined only at point 0 and five days after the first dose of rhTSH), but Karga et al. [20] observed a significant decrease in calcium concentration after administration of rhTSH compared to the control group (day one and day two) and to the baseline values (day 1), as well as an increase in PTH concentration on day five (compared to control and baseline values). It is worth adding that the calcium values observed by them were still within the normal range, which was probably due to the proper function of the parathyroid glands. These authors, like us, drew attention to the relationship between PTH and TSH in the study group, however, the correlation was very low (r = 0.142) and practically statistically insignificant (*p*~0.07) despite the relatively large amount of data (*n* = 160). It should be noted that the analyzed values came from different measurement points (the values at the different time points). The consistency of the results obtained by us is also proven by the confirmed relationship between PTH concentration (point 5) and ∆TSH.

This information is very important at least for two reasons:

1. PTH is one of the most important regulators of calcium-phosphate homeostasis, and considering bone as an effector of its action, it also has a huge impact on bone turnover. The increase in PTH can result from many factors, but the most important are vitamin D deficiency, renal failure, and age. Unfortunately, we did not find a correlation between PTH and 25(OH)D in our research (we assume that a relatively small number of respondents is important here), but the lack of a relationship does not mean that PTH does not reflect the adequacy of 25(OH)D concentration in individual subjects. We believe that the adequate level of vitamin D in the body is not only confirmed by the level of 25(OH)D [27], but also by other parameters of calcium and phosphate metabolism, including PTH. Nevertheless, the mean concentration of 25(OH)D observed in our cohort corresponds to a moderate vitamin D deficiency and may increase the baseline PTH concentration and, consequently, modulate the effect of rhTSH on bone.
2. Healthy parathyroid glands that secrete PTH in response to fluctuating ionized calcium levels are the most important regulators of calcium-phosphate homeostasis. Therefore, their efficiency is a guarantee of maintaining a constant, correct calcium concentration. Unfortunately, surgical treatment of the thyroid gland, especially radical ones (e.g., total thyroidectomy for DTC), is a common cause of hypoparathyroidism, so in such patients, administration of rhTSH may cause hypocalcemia. It is to be recalled that in our study the inclusion criterion was normal parathyroid function. However, if hypocalcemia happens at all, it is usually asymptomatic. It results from our own observations with the administration of rhTSH in the follow-up of DTC patients, as well as from the lack of such descriptions in the literature. Nevertheless, the potential pathomechanism of such hypocalcemia should remain in the minds of doctors dealing with such treatment.

Bellido et al. [13] found that chronic elevation of PTH in mice reduced the expression of sclerostin by osteocytes, while Keller and Kneissel [28] proved that PTH directly inhibits transcription in vivo and in vitro in mice, suggesting that SOST regulation may play a role in mediating PTH action in bone. In our studies, we did not find a relationship between the baseline PTH concentration and sclerostin both before and after rhTSH administration, however, we noticed that ∆sclerostin negatively correlated with PTH value both at point 0 (r = −0.431, *p* = 0.0195) and at point 5 (r = −0.405, *p* = 0.0293). It was similar in the case of the correlation between PTH (point 0) and ∆β-CrossLaps (r = −0.644, *p* < 0.005), but no such relationship was found in the case of PTH (point 0) and ∆osteocalcin (r = −0.299, *p* = 0.115). These observations confirm the importance of the baseline PTH concentration on bone metabolism as a major regulator of bone formation and resorption.

When discussing the influence of exogenous TSH on bone metabolism in the context of the initial PTH concentration, one should also mention the paper of Saracyn et al. [29], who proved that administration of rhTSH in patients after total thyroidectomy for DTC worsens renal cortical perfusion and renal function. The cited authors state that renal dysfunction in these patients may result from the direct action of TSH via the TSH receptors localized in small renal vessels and different segments of renal glomeruli, independent of thyroid hormone concentrations.

## 5. Conclusions

On the basis of our results, it can be concluded that:

1. rhTSH inhibits bone turnover, therefore TSH—in a manner independent of thyroid hormones—has a qualitative protective effect on bone metabolism.
2. Baseline PTH affects the magnitude of the increase in TSH and the degree of decrease in sclerostin and β-CrossLaps, therefore factors that affect the baseline PTH (e.g., age, vitamin D deficiency, renal failure) may play a role in the effect of TSH on bone but may also regulate changes in the concentration of sclerostin and other bone markers and through such a mechanism it can regulate processes of bone formation and resorption.
3. By the inhibition of bone metabolism, TSH can reduce calcium levels, which may be important when administering rhTSH to patients with postoperative hypoparathyroidism.

## Figures and Tables

**Table 1:** Descriptive data for investigated patients.

		*n*	Mean ± SD (Min–Max)
Group		29	
Female/Male		26/3	
Age [years]			52.4 ± 13.9 (30–86)
BMI [kg/m^2^]			28.0 ± 6.8 (19.3–52.2)
TNM staging system			
T	pT1 (pT1a/pT1b/pT1m)	23 (17/2/4)	
	pT2	4	
	pT3	1	
	pT4	1	
N	N0/N1	27/2	
M	M0	29	
L-T4 treatment			
	Substitutive	18	
	Incomplete TSH suppression (TSH: 0.1–0.4 mIU/L)	11	
Tg [ng/mL]			
	<0.1 (below detection limit) Detectable	17	
		12	0.68 ± 0.62 (0.13–2.0)

SD—standard deviation; BMI—body mass index; L-T4—L-thyroxine; Tg—thyroglobulin.

**Table 2:** The values of investigated parameters before (point 0) and after (point 5) rhTSH administration.

	Reference Range *	Mean ± SD	
		before (0)	after rhTSH (5 Days)
TSH [mIU/L]	0.4–4.2	0.52 ± 1.31	28.62 ± 16.41
FT4 [ng/mL]	0.83–1.7	1.99 ± 0.95	2.08 ± 0.86
FT3 [pg/mL]	2.6–4.4	3.14 ± 0.63	3.14 ± 0.72
Ca [mmol/L]	2.1–2.55	2.28 ± 0.18	2.24 ± 0.12
P [mmol/L]	0.81–1.45	1.33 ± 0.4	1.37 ± 0.49
Creatinine [mg/dL]	0.52–1.04	0.75 ± 0.18	N/D
GFR [mL/min/1.73 m^2^]		88.65 ± 18.29	N/D
PTH [pg/mL]	15–65	43.12 ± 22.42	41.83 ± 28.02
25(OH)D [ng/mL]	<20—deficiency 20–29 suboptimal 30–50 optimal 50–100 more than optimal >100—toxic	17.83 ± 7.77	N/D
Osteocalcin [ng/mL]	Women before menopause: 11–43 Women after menopause: 15–46 Men (18–30 y): 24–70 (30–50 y): 14–42 (>50 y): 14–46	24.88 ± 10.84	26.32 ± 11.68
β-CrossLaps [ng/mL]	Women before menopause: <573 Women after menopause <1008 Men (30–50 y): <584 (50–70 y) <704 (>70 y) <854	0.45 ± 0.28	0.48 ± 0.29
Sclerostin [pmol/L]	N/D	32.83 ± 20.12	32.72 ± 16.12
24-h urinary Ca [mmol]	2.5–7.5	3.49 ± 2.45	3.58 ± 2.35
24-h urinary P [mmol]	12.9–42	22.84 ± 9.87	21.99 ± 6.30

SD—standard deviation; N/D—no data; *—reference values used by local laboratory unit, y—years.

## Data Availability

Data supporting the reported results are available on request from the authors (A.Z. or A.L.).

## Abbreviations

^131^I	radioiodine;
25(OH)D	25-hydroxyvitamin D;
ATA	American Thyroid Association;
BALP	bone alkaline phosphatase;
BMD	bone mineral density;
BMI	body mass index;
Ca	calcium;
CrossLaps	C-telopeptides of type-1 collagen;
DTC	differentiated thyroid cancer;
GFR	glomerular filtration rate;
i.m.	intramuscular;
L-T4	L-thyroxine;
OPG	osteoprotegerin;
P	phosphate;
PINP	N-terminal propeptide of type-1 procollagen;
PTH	parathormon;
RANK	receptor of nuclear factor-κB;
RANKL	receptor of nuclear factor-κB ligand;
rhTSH	recombinant human thyrotropin;
β-CrossLaps	crosslinked isomerized type I collagen fragments;
Tg	thyroglobulin;
TSH	thyrotropin;
TSH-Rs	TSH receptors;
U.CTx	C-terminal telopeptide of type I collagen in urine;
U.NTx	N-terminal telopeptide of type I collagen in urine
